# Two Distinct Approaches for CRISPR-Cas9-Mediated Gene Editing in Cryptococcus neoformans and Related Species

**DOI:** 10.1128/mSphereDirect.00208-18

**Published:** 2018-06-13

**Authors:** Ping Wang

**Affiliations:** a Department of Pediatrics, Louisiana State University Health Sciences Center, New Orleans, Louisiana, USA; b Department of Microbiology, Immunology, and Parasitology, Louisiana State University Health Sciences Center, New Orleans, Louisiana, USA; Carnegie Mellon University; Yonsei University; Clemson University

**Keywords:** Gβ-like/RACK1 protein, homologous recombination-mediated gene editing, pCnCas9:U6-gRNA, ribonucleoprotein complex

## Abstract

Cryptococcus neoformans and related species are encapsulated basidiomycetous fungi that cause meningoencephalitis in individuals with immune deficiency. This pathogen has a tractable genetic system; however, gene disruption via electroporation remains difficult, while biolistic transformation is often limited by lack of multiple genetic markers and the high initial cost of equipment. The approach using clustered regularly interspaced short palindromic repeats (CRISPR) and CRISPR-associated protein 9 (Cas9) has become the technology of choice for gene editing in many organisms due to its simplicity, efficiency, and versatility. The technique has been successfully demonstrated in C. neoformans and Cryptococcus deneoformans in which two DNA plasmids expressing either the Streptococcus pyogenes *CAS9* gene or the guide RNA (gRNA) were employed. However, potential adverse effects due to constitutive expression and the time-consuming process of constructing vectors to express each gRNA remain as a primary barrier for wide adaptation. This report describes the delivery of preassembled CRISPR-Cas9-gRNA ribonucleoproteins (RNPs) via electroporation that is able to generate edited mutant alleles. RNP-mediated CRISPR-Cas9 was used to replace the wild-type *GIB2* gene encoding a Gβ-like/RACK1 Gib2 protein with a *gib2*::*NAT* allele via homologous recombination in both C. neoformans and C. deneoformans. In addition, a DNA plasmid (pCnCas9:U6-gRNA) that expresses both Cas9 and gRNA, allowing for convenient yet low-cost DNA-mediated gene editing, is described. pCnCas9:U6-gRNA contains an endogenous U6 promoter for gRNA expression and restriction sites for one-step insertion of a gRNA. These approaches and resources provide new opportunities to accelerate genetic studies of *Cryptococcus* species.

**IMPORTANCE** For genetic studies of the *Cryptococcus* genus, generation of mutant strains is often hampered by a limited number of selectable genetic markers, the tedious process of vector construction, side effects, and other limitations, such as the high cost of acquiring a particle delivery system. CRISPR-Cas9 technology has been demonstrated in *Cryptococcus* for genome editing. However, it remains labor-intensive and time-consuming since it requires the identification of a suitable type III RNA polymerase promoter for gRNA expression. In addition, there may be potential adverse effects caused by constitutive expressions of Cas9 and gRNA. Here, I report the use of a ribonucleoprotein-mediated CRISPR-Cas9 technique for genome editing of C. neoformans and related species. Together with the custom-constructed pCnCas9:U6-gRNA vector that allows low-cost and time-saving DNA-based CRISPR-Cas9, my approach adds to the molecular toolbox for dissecting the molecular mechanism of pathogenesis in this important group of fungal pathogens.

## Abstract (precis)

For genetic studies of the *Cryptococcus* genus, generation of mutant strains is often hampered by a limited number of selectable genetic markers, the tedious process of vector construction, side effects, and other limitations, such as the high cost of acquiring a particle delivery system. CRISPR-Cas9 technology has been demonstrated in *Cryptococcus* for genome editing. However, it remains labor-intensive and time-consuming since it requires the identification of a suitable type III RNA polymerase promoter for gRNA expression. In addition, there may be potential adverse effects caused by constitutive expressions of Cas9 and gRNA. Here, I report the use of a ribonucleoprotein-mediated CRISPR-Cas9 technique for genome editing of C. neoformans and related species. Together with the custom-constructed pCnCas9:U6-gRNA vector that allows low-cost and time-saving DNA-based CRISPR-Cas9, my approach adds to the molecular toolbox for dissecting the molecular mechanism of pathogenesis in this important group of fungal pathogens.

## INTRODUCTION

Cryptococcus neoformans and Cryptococcus deneoformans are encapsulated basidiomycetous fungal pathogens (1) that cause often-fatal cryptococcosis in immunocompromised individuals (2). Infections currently afflict approximately one million people worldwide, with fatalities near 624,700 annually (3) (also reviewed by Idnurm and Lin [4]). Virulence of *Cryptococcus* is a multifaceted trait, with the ability to grow at body temperature, the elaboration of the melanin pigment and a polysaccharide capsule, and the presence of the MATα mating type all contributing to its ability to cause the disease. Development and adaptation of advanced genetic tools such as gene disruption and RNA interference, as well as the completed sequencing and annotation of C. neoformans and C. deneoformans genomes, have greatly accelerated the efforts to understand the molecular mechanism of pathogenesis for this fungus (5, 6). Liu and colleagues have also generated a partial library of signature-tagged gene-specific mutants that could help to examine systemically molecular events leading to fungal pathogenesis. However, limitations such as the availability of only three dominant selection markers (hygromycin, nourseothricin, and G418 resistance), the labor-intensive construction of individual mutant alleles, biases in insertion sites, and chromosomal rearrangements, as well as difficulties for whole gene replacement or large construct insertions, are currently hindering research efforts.

Over the past several years, advanced methodology that employs meganucleases, zinc finger nucleases (ZFNs), transcription activator-like effector nucleases (TALENs), and clustered regularly interspaced short palindromic repeat (CRISPR)–CRISPR-associated nuclease 9 (Cas9) have been developed to allow highly efficient and precise genome editing or alterations (7, 8). Indeed, the CRISPR-Cas9 system has been recently demonstrated in C. neoformans (9) and C. deneoformans (10) for generation of gene-specific mutations. While these studies reported the use of a modified version of the Cas9 endonuclease from Streptococcus pyogenes, Arras and colleagues utilized a ribozyme-based technique to express guide RNA (gRNA), whereas Wang et al. utilized an endogenous U6 promoter from C. neoformans to drive the expression of gRNA. Constitutive expression of Cas9 and gRNA resulted in edited mutant alleles in C. neoformans (9) and C. deneoformans (10). These studies demonstrated the feasibility of implementing the CRISPR-Cas9 methodology for this fungus. In addition, Arras and colleagues reported that the resulting mutants showed no adverse side effects on phenotypes, including animal virulence (9). Nevertheless, the possibility that the delivered DNA integrates into the genome to cause side effects, including potential off-target cutting, remains (11–13). To circumvent this potential roadblock, Wang and colleagues designed the plasmid DNA to potentially allow the removal of the inserted Cas9 gene following gene editing (10). Very recently, a DNA-based CRISPR-Cas9 expression system specifically designed for transient expression has also been developed for this group of fungi (14).

In order to mitigate the aforementioned negative effects and reduce efforts spent on constructing vectors expressing Cas9 and each gRNA, DNA-free CRISPR-Cas9-mediated gene editing has been recently demonstrated in various systems, including several pathogenic fungi. In this methodology, *in vitro*-preassembled CRISPR-Cas9 ribonucleoproteins (RNPs) were delivered using electroporation (15, 16). This RNP-mediated genome editing technique has been successfully applied to *Candida* species (17, 18) and Aspergillus fumigatus (19). This approach holds a great appeal for genetic studies of C. neoformans and related species, because (i) gene disruption or other genome modifications necessitates a high-cost biolistic device (gene gun) since electroporation shows extremely low gene disruption efficiency (20), albeit the efficiency has been improved somewhat (21), (ii) constitutively activated *CAS9* and *gRNA* genes in the genome remain a concern, and (iii) RNP-mediated gene editing offers a true transient expression system. For this reason, I tested and demonstrated the feasibility of CRISPR-Cas9-mediated genome editing in the form of the RNP complex delivered by electroporation in C. neoformans and C. deneoformans. The resulting regenerated strains showed specifically targeted gene replacement at relatively high frequency. In addition, I also constructed the vector pCnCas9:U6-gRNA, which simultaneously expresses both Cas9 endonuclease and gRNAs: engineered restriction enzyme sites in pCnCas9:U6-gRNA also permit a simple insertion event for expression of specific gRNA sequences, and the pCnCas9:U6-gRNA plasmid was shown to induce gene editing by both electroporation and biolistic transformation in the C. neoformans strain that was tested.

## RESULTS

### Designs for CRISPR-Cas9 RNP-mediated GIB2 gene disruption.

**FIG 1:**
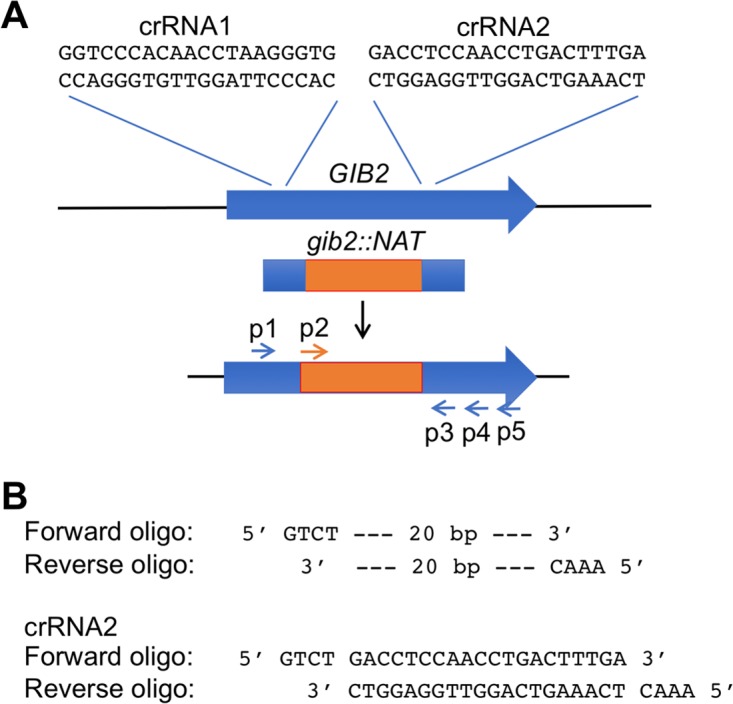
Illustration for the design of two crRNAs for the replacement of the *GIB2* gene with the mutant *gib2*::*NAT* allele. (A) A 20-nt sequence close to the 5′ side (crRNA1) and another close to the 3′ side (crRNA2) of the coding region. The 20-nt sequences are followed by the protospacer adjacent motif (PAM) site necessary for CRISPR-Cas9-directed DNA restriction. p1, PW166; p2, PW610; p3, PW2055; p4, PW277; p5, PW167. (B) A design example of 20-nt gRNA sequences plus 4-nt linkers.

The CRISPR-Cas9 system utilizes RNA-guided nuclease activity to introduce sequence-specific double-strand breaks (DSBs) and targeted genome editing. The system usually involves the use of two components: a Cas9 endonuclease and gRNA consisting of a fusion of a CRISPR guide RNA (crRNA) and a fixed transactivating crRNA (tracrRNA). In the DNA-free ribonucleoprotein complex, the Cas9 protein and transcribed gRNAs are assembled *in vitro* and delivered into the cells via lipofection or electroporation. The 20 nucleotides (nt) at the 5′ end of the gRNA direct Cas9 to a specific target DNA target site. To test the feasibility of this method, I opted to test gene replacement via homologous recombination in C. neoformans and C. deneoformans. I chose the *GIB2* gene that encodes an atypical Gβ-like/RACK1 protein, Gib2, because this gene is highly conserved between C. neoformans and related species and because of the availability of the *gib2*::*NAT* mutant allele from our previous studies (22–24). I also decided to design crRNAs for *GIB2* replacement with one 20-nt sequence close to the 5′ side and another close to the 3′ side of the coding region (Fig. 1). All 20-nt sequences were adjacent to a protospacer adjacent motif (PAM) site in the genome that is necessary for CRISPR-Cas9-directed restriction.

### RNP-mediated CRISPR-Cas9 gene editing via electroporation.

The purified Cas9 protein, specific crRNAs, and the universal tracrRNA, obtained from a commercial source (Integrated DNA Technologies, Inc., Coralville, IA), were mixed to assemble the ribonucleoprotein complex following the protocol provided by the manufacturer and others with minor modifications (17–19). crRNAs and the tracrRNA were mixed in a microcentrifuge tube prior to the addition of Cas9. The reaction mixture was then incubated at 30°C for 5 min. C. neoformans cells were prepared as previously described by Lin et al. and Wickes et al. (25, 26). Despite various treatments and concentrations of LiCl and dithiothreitol (DTT) for optimization, 1 mM DTT treatment of between 30 and 60 min yielded consistent results. Both the exponential method (0.450 kV, 125 µF, and 500 Ω) and a time constant protocol (1.8 to 2.0 kV, 5 ms) were used for electroporation. Following transformation, cells were recovered by incubation in liquid yeast extract-peptone-dextrose (YPD) at 30°C for 90 min and spread on YPD medium containing nourseothricin (70 µg/ml).

**FIG 2:**
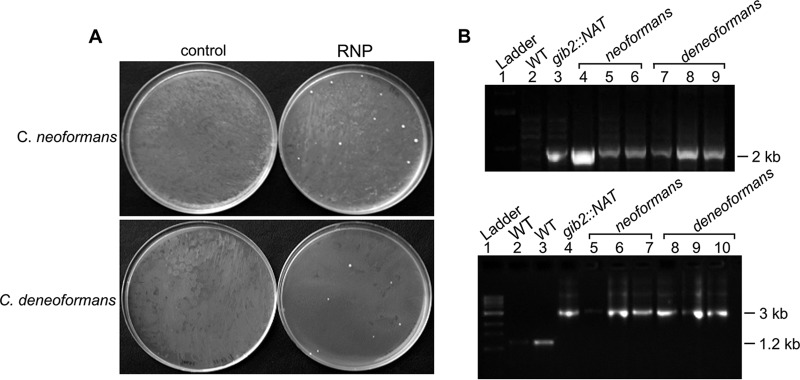
Ribonucleoprotein complex (RNP)-mediated CRISPR-Cas9 gene replacement achieved by electroporation. (A) The ribonucleoprotein complexes containing the Cas9 endonuclease, crRNA1 and crRNA2, and the *gib2*::*NAT* fragment were assembled prior to mixing with C. neoformans and C. deneoformans cells, respectively. Following electroporation (exponential protocol), cells were mixed with YPD with 0.5 M sorbitol for 90 min with shaking (×150 rpm). Plates were allowed to incubate for at least 3 days or until colonies appear. Images show the results of such a transformation following a 3-day incubation at 30°C. C. neoformans is shown in the top panel and C. deneoformans in the bottom panel. (B) Primers PW610 and PW2055 were used to amplify the ~2.0-kb partial *gib2*::*NAT* allele from the RNP-mediated CRISPR-Cas9 gene replacement strains as illustrated in Fig. 1. Upper panel: lane 1, 1-kb ladder; lane 2, H99; lane 3, the original *gib2*::*NAT* knockout mutant (23); lanes 4 to 6, RNP transformants of C. neoformans (H99); lanes 7 to 9, RNP transformants of C. deneoformans (JEC21). Primers PW166 and PW277 were used to amplify the ~3.0-kb *gib2*::*NAT* allele. Lower panel: lane 1, 1-kb ladder; lane 2, H99; lane 3, JEC21; lane 4, the original *gib2*::*NAT* knockout mutant (23); lanes 5 to 7, RNP transformants of C. neoformans (H99); lanes 8 to 10, RNP transformants of C. deneoformans (JEC21).

The *GIB2* gene encoding the atypical Gβ-like/RACK1 Gib2 protein is highly conserved between C. neoformans strain H99 and C. deneoformans strain JEC21, sharing high nucleotide sequence (96.5%) and amino acid sequence (100%) identities. Identical 20-bp gRNAs plus a 3-bp PAM were designed so that the crRNA could be used for both species (Fig. 1). With the ~3.2-kb *gib2*::*NAT* allele as a donor DNA, I obtained 26 transformants from two plates (cuvettes) for C. neoformans (H99) and 17 transformants for C. deneoformans (JEC21) (Fig. 2A). No colonies were found in the control in which only donor DNA was used for electroporation (Fig. 2A). To test if the transformants were putative RNP-mediated *gib2*::*NAT* mutant strains, genomic DNA was extracted from six candidates each, and PCR amplification analysis was performed. Primers PW610 and PW2055 amplified a partial ~2-kb *gib2*::*NAT* allele from three tested *NAT*^R^ transformants of each species tested, while PW166 and PW277 amplified the ~1.2-kb fragment from the wild-type strain (H99) and the ~3-kb fragment from the transformants (Fig. 2B). This result indicated that both C. neoformans and C. deneoformans strains are amendable to genetic manipulation by RNP-mediated CRISPR-Cas9 technology.

### Construction and testing of pCnCas9:U6-gRNA for DNA-based CRISPR-Cas9 gene editing.

**FIG 3:**
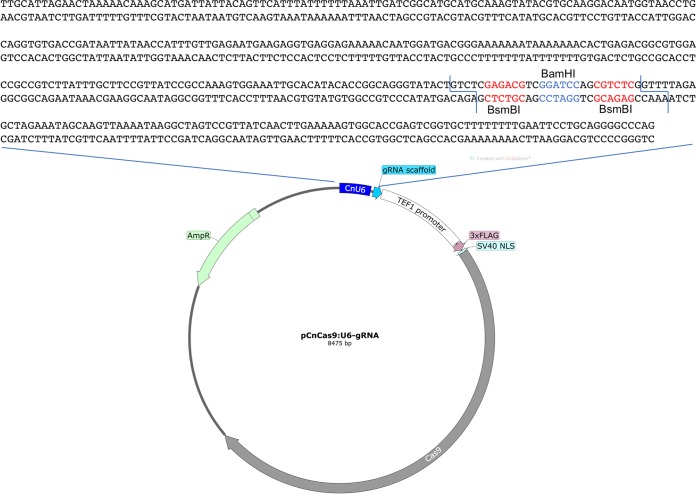
Schematic representations of the plasmid pCnCas9:U6-gRNA and the design for gRNA expression driven by the C. deneoformans U6 RNA polymerase III promoter in pCnCas9:U6-gRNA. Key features of pCnCas9:U6-gRNA are depicted. Two BsmBI/Esp3I restriction enzyme sites were built into the sequence following the U6 promoter. This construct will no longer be cleavable by BsmBI/Esp3I once the annealed 20-nt gRNA sequence is inserted.

Prior to testing RNP-mediated CRISPR-Cas9 for C. neoformans, I have also focused on simplification of the steps needed for DNA-based CRISPR-Cas9 gene editing in C. neoformans. To achieve this, a custom vector, pCnCas9:U6-gRNA containing both the *CAS9* gene expressing Cas9 and the gRNA-expressing cassette, was constructed (Fig. 3). The vector was based on pmCas9:tRNAp-gRNA that contains a 75-nt Gln tRNA from the rice false smut fungus Ustilaginoidea oryzae that exhibits a polymerase III (Pol III) RNA polymerase promoter activity and nuclear localization signal (NLS)-3×FLAG-Cas9 driven by a translation elongation factor (TEF) promoter (Keiling Zhong and Zhengguang Zhang, unpublished data). Two BsmBI/Esp3I restriction sites were introduced to enable the insertions of an annealed 20-nt gRNA primer plus a 4-nt linker sequence before the gRNA scaffold. This design is similar to pX330 described by Cong and colleagues (27). However, we could not obtain any transformants following multiple tries, suggesting that the U. oryzae promoter sequence may not be functional in *Cryptococcus* (data not shown). I therefore replaced this sequence with a U6 promoter identified by Wang and colleagues (10).

**FIG 4:**
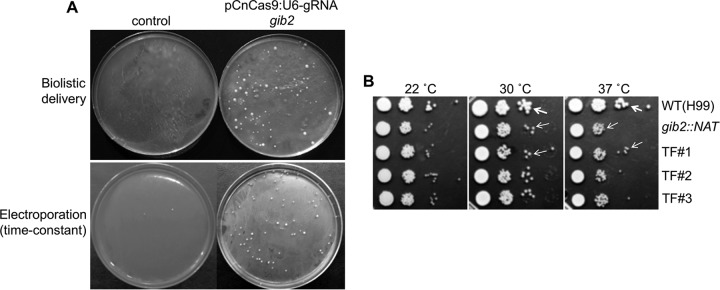
pCnCas9:U6-gRNA plasmid-mediated CRISPR-Cas9 gene replacement. (A) Two pCnCas9:U6-gRNA constructs containing *gib2-*gRNA sequences targeting the 5′ and 3″ sites of the *GIB2* gene were mixed with the gold particles in the presence of the ~3.2-kb *gib2*::*NAT* mutant fragment donor DNA and delivered into the H99 strain via the biolistic delivery apparatus (top panel) and electroporation (bottom panel, time-constant protocol). Plates were incubated at 30°C for 3 days (as shown) or until colonies emerged. (B) The *gib2*::*NAT* gene replacement transformants exhibit attenuated thermal sensitivity like the original *gib2*::*NAT* mutant strain (23). Thin arrows point to smaller colonies, while thick arrows point to the larger colonies of wild-type strain H99.

The 20-nt gRNA primer sequences were annealed and ligated into pCnCas9:U6-gRNA digested with BsmBI/Esp3I. Ligation products were identified by the loss of the BsmBI/Esp3I (and BamHI) site. Sequence data for gRNA design and expression within pCnCas9:U6-gRNA are shown in Fig. 3. Two pCnCas9:U6-gRNA plasmids expressing the *gib2* gRNA sequences (the same as in the RNP method) were introduced into H99 by electroporation (5 µg) or biolistic transformation (2 µg), in the presence of donor DNA (~3.2-kb *gib2*::*NAT* fragment). For biolistic transformation, ~114 nourseothricin-resistant colonies were obtained, in contrast to none from the donor DNA-only control plate (Fig. 4A, upper panel), while ~104 transformants were generated by electroporation, significantly more than the control (Fig. 4A, lower panel). Putative mutant strains were streaked onto fresh medium, and genomic DNA was extracted from six putative transformants obtained by biolistic transformation for verification by PCR amplification. PCR amplification with primers PW166 and PW2055 yielded the presence of an ~3-kb fragment containing the *gib2*::*NAT* allele in the transformants, while the wild-type *GIB2* allele was present only in the wild-type strain, similar to that shown in Fig. 2B (data not shown). In addition, testing of randomly selected 7 transformants all displayed slight but noticeably smaller colony sizes indicating thermal sensitivity, similar to that of the original *gib2*::*NAT* mutant strain (Fig. 4B, three transformants shown) and in comparison to the wild-type strain H99 (Fig. 4B, pointed to by thick arrows) (23).

## DISCUSSION

The CRISPR-Cas9 system is a strategy that utilizes RNA-guided nuclease activity to introduce sequence-specific double-strand breaks (DSBs) into DNA. This approach that can create targeted genome edits has revolutionized genetic studies by providing a convenient method for inducing targeted deletions, insertions, and precise sequence changes in a broad range of many cell types and organisms. These species include human cell lines, mice, zebra fish, Caenorhabditis elegans, plants, and bacteria (reviewed in reference 7). The method has also been demonstrated in many fungi, including unicellular yeasts and filamentous fungi. We have been testing the application of this technology for the basidiomycete yeast C. neoformans through the construction of a pGMC300-based vector that expresses the bacterial *CAS9* gene isolated from the DNA plasmid pCas (28). Because this system requires a type III RNA polymerase (Pol III) promoter to express the gRNAs, which are rarely identified from C. neoformans or C. deneoformans, we undertook a lengthy process of attempting to identify endogenous Pol III promoters. Toward this goal, we have performed transcriptome sequencing (RNA-Seq) analysis of secreted small noncoding RNAs from the C. deneoformans JEC21 strain as they are likely transcribed from Pol III promoters. We have obtained ~200,000 secreted small noncoding RNAs (Wang, unpublished data), but the task of mining for such a promoter(s) remains daunting. Since a U6 promoter was identified earlier (10), I opted to replace the U. oryzae tRNA with this promoter. In the resulting plasmid, pCnCas9:U6-gRNA, specific 20-nt gRNAs can be synthesized and easily inserted, thus cutting experimental costs and time. Over the course of these studies, I found that the RNP-mediated CRISPR-Cas9 technique is also suitable for gene manipulations in *Cryptococcus* species. Despite the high cost associated with purchasing of the enzyme, crRNA, and tracrRNA, it remains desirable due to its ease of operation, the transient presence of Cas9 and gRNA, and convenient delivery via electroporation. While the ability to introduce mutations due to nonhomologous end joining (NHEJ), in which double-strand break ends are directly ligated without the need for a homologous template, remains to be tested, these two different approaches should complement each other nicely in introducing gene replacements by homology recombination.

In the application of the RNP-mediated gene edits of Aspergillus fumigatus, Al Abdallah and colleagues found that the homologous sequence of ~50 nt is sufficient to induce homology-directed repair (HDR) in the CRISPR-Cas9-mediated genome editing (19). A recent study by Fan and colleagues reported that while ~50 nt cannot induce HDR, it was able to introduce DNA breakpoints (14). The difference is not surprising given the divergence of the two different fungal species. I designed two crRNAs for the *GIB2* gene since the previously generated *gib2*::*NAT* allele of C. neoformans could also be used as a donor for gene replacement in C. deneoformans. Interestingly, the same *gib2*::*NAT* mutant allele could also be included in the RNP-mediated transformation of C. gattii/Candida deuterogatti as a donor DNA with the same 5′ crRNA (crRNA1) but a different 3′ crRNA (crRNA3) (Table 1). Although the experiment was not a quantitative comparison, two transformants were obtained, which may suggest that the RNP complex may also be used to edit gene structures in C. gattii/C. deuterogatti.

**TABLE 1:** Primers used in this study

Primer	Sequence^a^
crRNA1	5′-GGTCCCACAACCTAAGGGTG-3′
crRNA2	5′-GACCTCCAACCTGACTTTGA-3′
crRNA3	5′-GACCTTCAACCCGACTTTGA-3′
PW166	5′-CTTCGTTCATCTTTCACTGTTC-3′
PW167	5′-ACGAGACGACTCTGATTCTGAC-3′
PW277	5′-GGTAGCAGCACAGAGCCAGTA-3′
PW610	5′-GCTGCGAGGATGTGAGCTGG-3′
PW2051	5′-AAGGTACCTTGCATTAGAACTAAAAACAAAGCATG-3′
PW2052	5′-GGAATTCAAAAAAAAGCACCGACTCGGTGCCACTTTTTCA AGTTGATAACGGACTAGCCTTATTTTAACTTGCTATTTCTAG CTCTAAAACCGAGACGCTGGATCCGACGTCTCGAGACAGT ATACCCTGCCGGTGTATGTGC-3′
PW2055	5′-GACGAGAGCGTTGATAACGTCT-3′
PW2059	5′-AAACCACCCTTAGGTTGTGGGACC-3′
PW2064	5′-GTCTGGTCCCACAACCTAAGGGTG-3′
PW2061	5′-AAACTCAAAGTCAGGTTGGAGGTC-3′
PW2065	5′-GTCTGACCTCCAACCTGACTTTGA-3′

a Four-nucleotide linkers are underlined.

This study has demonstrated that the CRIPSR-Cas9 RNP complex delivered directly by electroporation is sufficient to induce gene editing in two main species of *Cryptococcus*. For reasons still not fully understood, gene disruption is difficult to achieve if DNA is introduced via electroporation with several selectable markers. Our findings open a door for many research labs that wish to employ gene disruption or other editing in their studies, but do not currently have access to a biolistic transformation apparatus. For those who do have access to biolistic transformation equipment, a previous report indicated that the RNP complex could also be delivered by biolistic transformation into the plant maize (16).

In summary, I have demonstrated that the CRISPR-Cas9 ribonucleoprotein complex can induce gene modification in two main species of *Cryptococcus* when introduced via electroporation. The presence of homologous sequences results in homologous recombination, making the technique suitable for genome editing. Together with pCnCas9:U6-gRNA for normal DNA-based CRISPR-Cas9-mediated gene editing, but with lower cost and the ease of gRNA insertion, these two approaches provide new avenues for others who wish to study molecular mechanisms of pathogenesis in *Cryptococcus*.

## MATERIALS AND METHODS

### Fungal strains and plasmid DNA.

Wild-type strains, including C. neoformans H99 (serotype A) and C. deneoformans JEC21 (serotype D) and C. gattii/C. deuterogattii WM268 (serotype B, VGIIa), were used in this study (29–31). All strains were cultured in yeast extract-peptone-dextrose (YPD) medium for 3 days at 30°C. Construction of the *gib2*::*NAT* mutant allele was previously described (19, 20). Briefly, the nourseothricin resistance gene cassette was inserted into the coding region of the *GIB2* gene that encodes the atypical Gβ/RACK1-like adaptor protein of Cryptococcus neoformans (22–24). The mutant allele was used as donor DNAs for the Cas9-mediated gene deletions described in this work. The *gib2*::*NAT* fragment was amplified by PCR with primers PW166 and PW167 and purified using the Qiagen gel extraction kit (Qiagen). The ~3.2-kb fragment contains the *NAT* cassette flanked by ~900-bp 5′ and ~800-bp 3′ homologous regions of the *GIB2* gene.

To replace the 75-nt U. oryzae tRNA sequence of pmCas9:tRNAp-gRNA with an endogenous U6 promoter, primers PW2051 and PW2052 were used to link the U6 promoter from C. deneoformans (10) with the BsmBI/Esp3I restriction sites followed by the tracrRNA sequence. The KpnI-EcoRI fragment was used to replace the original fragment within pmCas9:tRNAp-gRNA resulting in pCnCas9:U6-gRNA. The plasmid and the sequence are available upon request while the deposition into public domains is in progress.

Construction of plasmids expressing Cas9 and *gib2-*specific gRNAs was made possible by an insertion of annealed, complementary 20-nt oligonucleotide gRNA oligonucleotides as gRNA sequence plus a 4-nt linker at the BsmBI/Esp3I site. Insertion of the gRNA sequence eliminates the BsmBI/Esp3I restriction enzyme site so that positive clones can be easily screened. Primer names and their sequences are listed in Table 1.

### Design of crRNA and preparation of the ribonucleoprotein complex.

To test the CRISPR-Cas9 ribonucleoprotein complex-mediated gene disruption, two Cas9-gRNA constructs were employed. First crRNA was designed to target the region closer to the 5′ end of the *Gib2* gene, while the second crRNA was designed to be outside the coding region near the stop codon (Fig. 1). The same crRNAs were used to target the *GIB2* gene of H99 (C. neoformans) and JEC21 (C. deneoformans). For C. gattii/C. deuterogattii, a new 3′ crRNA (crRNA3) was needed, while the crRNA targeting 5′ of the *GIB2* gene (crRNA1) remains the same. gRNA sequences were designed using the resources at Integrated DNA Technologies, Inc. (IDT; Coralville, IA).

To prepare the ribonucleoprotein complex, two crRNAs were hybridized to the equal molar amounts of tracrRNA in nuclease-free duplex buffer (100 mM KCH_3_COO, 30 mM HEPES, pH 7.5 [IDT]) to ~20 µM. The mixture was annealed by heating at 94°C for 5 min and then slowly cooling down to room temperature (20°C). The resulting gRNA was used immediately or stored at −20°C. To generate the Cas9 ribonucleoprotein complex, 1 µl of gRNA, 8 µl of nuclease-free reaction buffer (150 mM KCl, 20 mM HEPES, pH 7.5), and 1 µl of diluted Cas9 (1 µg/µl in reaction buffer [IDT]) were mixed. The mixture was incubated at 30°C for 5 min to allow ribonucleoprotein complex formation.

### Electroporation.

Electroporation-mediated transformation of *Cryptococcus* species was performed in a Bio-Rad electroporation system (Gene Pulser Xcell) according to a previously published protocol (21, 25). Briefly, fungal cells grown overnight in liquid YPD were used to start fresh cultures the next day. When cultures reached an OD of ~1.0, the cells were precipitated and washed three times with distilled water. Cells were washed once in electroporation buffer (EB: 10 mM Tris-HCl, 1 mM MgCl_2_, 270 mM sucrose, pH 7.5) and resuspended in EB with 1 mM DTT for 30 min. Cells were washed again, resuspended in 1/10 of the original volume of EB, and incubated on ice until use. For electroporation, we mixed the ribonucleoprotein complexes described above and 2 µg of *gib2*::*NAT* donor DNA with 100 µl fungal cells on ice. Electroporation was performed within 5 min of mixing using either the exponential protocol (0.450 kV, 125 µF, and 500 Ω) or the time constant protocol (1.8 to 2.0 kV, 5 ms). Following electroporation, 1 ml of cold YPD with 0.5 M sorbitol and the contents of the cuvette were transferred to a 1.5-ml microcentrifuge tube for recovery by incubation at 30°C for 90 min with shaking (150 rpm). Cells were then pelleted and plated onto YPD plates (one cuvette to one plate) with nourseothricin (70 µg/ml). The plates were incubated at 30°C for 3 days or until colonies emerged.

### Oligonucleotide annealing and biolistic transformation.

To anneal complementary oligonucleotide primers prior to ligation into pCnCas9:U6-gRNA, oligonucleotides were dissolved in the aforementioned nuclease-free duplex buffer. Complementary primer pairs were heated by being submerged in boiling water for 4 min and then allowed to gradually cool down to room temperature. Annealed oligonucleotides (25 nmol) were diluted 50× with nuclease-free water prior to ligation.

For biolistic transformation, two pCnCas9:U6-gRNA plasmid constructs, each expressing an individual *gib2* gRNA, and donor DNA (*gib2*::*NAT*) were mixed with gold particles. Processing of the particles and biolistic transformation were carried out as described previously (32). For both electroporation and biolistic transformation, the 3.2-kb *gib2*::*NAT* fragment alone was used as a control.

### Verification of *gib2*::*NAT* mutants.

Putative transformants were streaked onto fresh selective medium and grown in liquid cultures for genomic DNA extraction. Primers PW166 and PW2055 were used in PCR amplification to generate an ~3-kb *gib2*::*NAT* fragment, while primers PW166 and PE167 were used to amplify the 1.7-kb wild-type *GIB2* allele. For phenotype verification, transformants and control strains were serially diluted and spotted onto YPD medium for testing at 20, 30, and 37°C as described previously (23).

### Accession number(s).

The complete nucleotide sequence of pCnCas9:U6-gRNA can be found in Fig. S1 and has also been deposited in GenBank under accession no. MH220818.

10.1128/mSphereDirect.00262-18.1 FIG S1  pCnCas9:U6-gRNA nucleic acid sequence. Download FIG S1, docx file, 0.02 MB. Copyright © 2018 Wang. 2018 Wang This content is distributed under the terms of the Creative Commons Attribution 4.0 International license.
