# Supplementary material for: Two Distinct Approaches for CRISPR-Cas9-Mediated Gene Editing in Cryptococcus neoformans and Related Species
Source: mSphere. 2018 Jun 13;3(3):e00208-18. doi: 10.1128/mSphereDirect.00208-18 (PMC6001613; doi:10.1128/mSphereDirect.00208-18)
Supplement: FIG S1 [file sph003182547sf1.docx]

Supplemental Figure 1. pCnCas9:U6-gRNA nucleic acid sequence^#^.

GGTACCTTGCATTAGAACTAAAAACAAAGCATGATTATTACAGTTCATTTATTTTTTAAATTGATCGGCATGCATGCAAAGTATACGTGCAAGGACAATGGTAACCTGCAGGTGTGACCGATAATTATAACCATTTGTTGAGAATGAAGAGGTGAGGAGAAAAACAATGGATGACGGGAAAAAAATAAAAAAACACTGAGACGGCGTGGACCGCCGTCTTATTTGCTTCCGTTATCCGCCAAAGTGGAAATTGCACATACACCGGCAGGGTATACTGTCTCGAGACGTCGGATCCAGCGTCTCGGTTTTAGAGCTAGAAATAGCAAGTTAAAATAAGGCTAGTCCGTTATCAACTTGAAAAAGTGGCACCGAGTCGGTGCTTTTTTTTGAATTCCTGCAGCCCGGGCCCAGCAAACGGTGGTCAAAGGATGGTTCAGATACAAATTAGCAACAGGCCAGGCTAGACGCGCGACTATCCACTGCGGCAAATGGTGAGCTGCAAGCAACGGTAAGATGTGACAGGACGAGCGGTGTGCCGGGAAAAAAATTGGAGGAGCGCAAAGCGGCGGCTGTCCCTCAGTGGTGCCCAAACGTTATCGATAGTACACCAAGCATGGGCAGTGAGCGGCTATACAGAGGGAATAATAGGCATATCGGCACGACTAGATTCGGTAGAAAGCATCGAAGAGCAATTCATTGAGCATATTATCACGTGGAATGCGATAGCTGTGGCCAGGTTGAGACACCGCAAGTGAAAGATACACACATAGATTCTCGATTCGAGCGGTTTGCCTCCGCCACCGCAGTGCATAGCAAGCAAAGAAACGACAGTTGGCTCATCATCCGTTACATCATTTTTTCTACTGGCTCCGCTCGGTGGGCTCCCAACGAAGCAGCAAAAAAGTGAGAGAAAAAAACTAGCTTGGCGGGGCAACAGAAGCTAGACCCTTTGGCTCGCTTAGTCAGTGCGCCCACTCACTCACACTCAAAAAGGCCACCCCTCCCGCACCCTCTTNCTCATCACCGTCTTCATACCACGGTTCGTCAAGCAATCGTATCTGGTAAGCTTTGACCTCCTCGAGCGGGCTCCACTTTGCTATTTCTTGGATCTGCTCTTTCTTTTCTCTCTACCTCTTTTTCTAACCTCTCTTCAGAAAGTTCAACCGTACTTCACTCAATCTTCCATACACAACCGTCGGCGCGCCATGGACTACAAGGACCACGACGGTGACTATAAGGACCACGACATCGACTATAAGGACGACGACGACAAGATGGCCCCCAAGAAGAAGCGTAAGGTCATGGACAAGAAGTACTCCATCGGTCTCGACATCGGAACTAATTCCGTGGGCTGGGCTGTCATCACTGATGAGTACAAGGTTCCTTCCAAGAAGTTTAAAGTTCTTGGGAACACCGATAGGCACAGTATTAAGAAAAACCTGATCGGGGCTCTCCTCTTTGACAGTGGCGAAACGGCGGAAGCAACCCGTCTTAAGCGCACCGCCCGTCGGCGGTACACGAGGAGGAAGAATAGAATTTGCTATCTCCAAGAGATCTTCTCCAATGAAATGGCCAAGGTCGATGATTCCTTTTTTCATCGGCTGGAGGAATCCTTCCTCGTCGAGGAGGACAAGAAGCATGAAAGGCATCCCATCTTCGGCAATATCGTCGATGAGGTCGCATATCACGAGAAATACCCGACAATCTACCATCTCCGCAAGAAGCTCGTGGACTCGACCGACAAGGCCGATCTCCGATTGATTTACTTGGCTCTGGCACATATGATCAAATTTAGGGGCCACTTCCTCATCGAGGGAGATCTCAACCCCGACAACTCGGACGTCGATAAACTCTTCATCCAACTTGTTCAAACTTACAACCAATTGTTCGAAGAGAACCCTATTAACGCCAGCGGCGTCGACGCAAAGGCCATTCTGTCTGCCAGACTCTCTAAGAGCAGACGACTTGAAAACCTTATCGCCCAACTCCCCGGCGAAAAGAAGAACGGCCTCTTTGGCAACCTCATCGCCCTCTCCTTGGGTCTGACCCCGAACTTTAAGTCCAACTTCGACTTGGCAGAGGATGCCAAGCTTCAGCTCTCAAAGGATACGTATGATGACGACCTCGACAACCTGCTCGCCCAGATCGGGGACCAGTACGCGGATCTCTTCCTGGCCGCTAAGAACCTGAGCGATGCGATTCTGCTGTCCGACATCCTTAGGGTTAATACCGAGATCACAAAGGCCCCCCTGTCTGCATCCATGATCAAACGCTACGACGAGCATCATCAAGATCTTACACTCCTTAAGGCTCTCGTCCGCCAACAACTGCCTGAGAAGTACAAGGAGATCTTCTTTGACCAATCAAAGAACGGGTACGCAGGTTACATCGACGGCGGCGCGTCCCAGGAGGAATTTTACAAGTTCATCAAGCCGATCCTCGAAAAGATGGACGGCACCGAGGAACTCTTGGTGAAGCTCAACAGGGAGGACCTCTTGCGAAAGCAGCGGACCTTCGATAATGGTTCCATCCCGCACCAGATCCACCTCGGCGAGTTCCATGCCATTCTCCGTCGACAGGAGGACTTCTATCCGTTTCTCAAGGACAATAGGGAAAAAATCGAGAAAATCCTCACCTTCCGCATCCCTTACTACGTCGGCCCGCTCGCCCGAGGCAACTCCCGCTTTGCTTGGATGACGCGAAAGTCGGAGGAGACCATCACCCCATGGAACTTCGAGGAGGTGGTCGACAAGGGAGCTTCCGCCCAGAGTTTTATCGAGAGAATGACTAATTTCGATAAGAACTTGCCTAACGAGAAGGTCCTTCCCAAACATTCACTCCTCTACGAGTATTTTACCGTTTACAACGAGCTGACCAAGGTCAAGTACGTCACCGAGGGAATGCGCAAGCCCGCTTTCCTCTCGGGCGAGCAGAAGAAGGCAATTGTTGACCTGCTCTTCAAGACGAACCGTAAGGTCACAGTGAAGCAGCTCAAGGAAGATTACTTCAAGAAAATCGAGTGTTTCGACAGCGTTGAGATTTCCGGTGTTGAGGACCGCTTCAACGCCAGCCTTGGCACTTACCACGATCTGCTCAAAATTATCAAGGATAAAGATTTTCTTGACAACGAANAGAATGAGGACATTCTTGAGGATATCGTCCTTACCCTCACTTTGTTCGAGGATCGCGAAATGATTGAAGAACGACTCAAAACCTACGCCCATCTTTTCGACGATAAGGTCATGAAACAGCTTAAACGTAGACGGTACACTGGGTGGGGGCGACTGTCCAGAAAGTTGATTAACGGAATCCGTGACAAACAAAGCGGTAAGACTATCTTGGACTTCCTCAAATCCGATGGATTCGCTAACCGCAACTTCATGCAACTCATCCACGACGATTCTCTCACATTCAAGGAGGATATTCAGAAGGCCCAGGTGTCTGGCCAGGGCGACAGTCTGCACGAGCATATTGCCAACCTCGCTGGGTCTCCAGCCATCAAAAAAGGGATTCTGCAAACAGTGAAAGTTGTCGATGAATTGGTCAAGGTTATGGGTAGGCACAAGCCCGAGAACATCGTTATCGAGATGGCGCGAGAGAATCAAACCACGCAGAAAGGTCAAAAGAACAGTAGGGAGCGCATGAAGAGAATTGAAGAGGGCATCAAGGAACTTGGGTCGCAGATCCTCAAGGAACATCCCGTGGAAAACACCCAGCTGCAGAATGAAAAGCTCTACCTGTACTATCTCCAAAACGGTCGGGACATGTACGTTGACCAGGAGCTGGATATCAACCGGCTCTCTGACTACGATGTCGATCATATCGTGCCTCAGTCCTTCCTCAAGGATGACTCCATCGACAATAAGGTCCTCACCCGATCAGACAAGAACCGTGGCAAGTCGGACAACGTCCCGTCCGAAGAGGTCGTGAAGAAGATGAAGAACTACTGGCGTCAGTTGCTCAACGCCAAGTTGATCACCCAACGGAAATTTGATAACCTGACCAAGGCCGAACGCGGCGGACTCAGTGAACTTGACAAGGCCGGCTTCATCAAACGTCAACTTGTTGAAACCCGCCAAATCACGAAGCACGTGGCGCAGATCTTGGATTCGCGGATGAACACCAAGTATGACGAAAACGATAAGCTCATTCGTGAAGTCAAAGTCATCACCCTTAAAAGCAAGCTTGTTAGCGACTTTCGCAAGGATTTCCAGTTTTACAAGGTTAGGGAGATCAACAACTACCATCACGCCCATGACGCGTATCTCAACGCAGTCGTCGGCACTGCACTTATCAAGAAGTATCCTAAGTTGGAATCCGAATTTGTTTATGGGGATTACAAGGTGTATGACGTCCGAAAGATGATTGCCAAGTCAGAACAGGAGATCGGGAAGGCCACAGCCAAATATTTCTTCTATTCCAACATCATGAATTTCTTCAAGACCGAGATTACTCTGGCGAACGGTGAGATTCGTAAGCGTCCTCTTATCGAGACCAATGGAGAGACAGGTGAAATCGTTTGGGATAAGGGGCGCGACTTCGCTACGGTCAGGAAGGTGCTGTCGATGCCTCAAGTGAACATCGTGAAGAACACCGAAGTCCAAACGGGTGGTTTCTCTAAGGAAAGTATTCTGCCTAAGCGAAACTCTGACAAACTGATTGCGCGAAAGAAAGACTGGGACCCAAAGAAGTATGGCGGCTTCGACTCCCCTACAGTTGCTTACTCTGTTCTCGTCGTTGCTAAGGTCGAGAAGGGAAAATCGAAGAAGCTCAAGAGTGTCAAGGAATTGCTCGGGATTACGATCATGGAGCGTAGCTCGTTCGAGAAGAACCCGATCGATTTTTTGGAGGCTAAGGGCTATAAGGAAGTCAAGAAAGACCTTATCATCAAATTGCCCAAGTACAGCCTGTTTGAACTTGAGAATGGTCGCAATCGTATGCTTGCTTCAGCTGGCGAACTCCAAAAGGGCAACGAACTCGCACTCCCTAGTAAATACGTCAACTTCCTCTATCTCGCATCACACTATGAGAAGCTCAAGGGATCTCCTGAGGACAATGAGCAAAAGCAATTGTTCGTGGAGCAACACAAGCACTATCTGGATGAGATTATTGAGCAAATCTCGGAGTTCTCCAAGCGCGTCATCCTGGCTGATGCCAACCTGGACAAGGTGCTCAGTGCCTATAATAAGCACCGCGATAAGCCGATCCGTGAGCAAGCCGAGAACATTATTCACCTCTTCACTCTGACCAATTTGGGAGCACCCGCGGCGTTCAAGTACTTCGACACCACCATCGATAGGAAAAGATATACCAGCACTAAAGAAGTGCTTGATGCCACGTTGATTCATCAGTCCATCACAGGTTTGTATGAGACCCGCATCGACCTGAGCCAGCTCGGCGGAGATTAGTTAATTAAACAATCAATCCATTTCGCTATAGTTAAAGGATGGGGATGAGGGCAATTGGTTATATGATCATGTATGTAGTGGGTGTGCATAATAGTAGTGAAATGGAAGCCAAGTCATGTGATTGTAATCGACCGACGGAATTGAGGATATCCGGAAATACAGACACCGTGAAAGCCATGGTCTTTCCTTCGTGTAGAAGACCAGACAGGAGCTCCAGCTTTTGTTCCCTTTAGTGAGGGTTAATTTCGAGCTTGGCGTAATCATGGTCATAGCTGTTTCCTGTGTGAAATTGTTATCCGCTCACAATTCCACACAACATACGAGCCGGAAGCATAAAGTGTAAAGCCTGGGGTGCCTAATGAGTGAGCTAACTCACATTAATTGCGTTGCGCTCACTGCCCGCTTTCCAGTCGGGAAACCTGTCGTGCCAGCTGCATTAATGAATCGGCCAACGCGCGGGGAGAGGCGGTTTGCGTATTGGGCGCTCTTCCGCTTCCTCGCTCACTGACTCGCTGCGCTCGGTCGTTCGGCTGCGGCGAGCGGTATCAGCTCACTCAAAGGCGGTAATACGGTTATCCACAGAATCAGGGGATAACGCAGGAAAGAACATGTGAGCAAAAGGCCAGCAAAAGGCCAGGAACCGTAAAAAGGCCGCGTTGCTGGCGTTTTTCCATAGGCTCCGCCCCCCTGACGAGCATCACAAAAATCGACGCTCAAGTCAGAGGTGGCGAAACCCGACAGGACTATAAAGATACCAGGCGTTTCCCCCTGGAAGCTCCCTCGTGCGCTCTCCTGTTCCGACCCTGCCGCTTACCGGATACCTGTCCGCCTTTCTCCCTTCGGGAAGCGTGGCGCTTTCTCATAGCTCACGCTGTAGGTATCTCAGTTCGGTGTAGGTCGTTCGCTCCAAGCTGGGCTGTGTGCACGAACCCCCCGTTCAGCCCGACCGCTGCGCCTTATCCGGTAACTATCGTCTTGAGTCCAACCCGGTAAGACACGACTTATCGCCACTGGCAGCAGCCACTGGTAACAGGATTAGCAGAGCGAGGTATGTAGGCGGTGCTACAGAGTTCTTGAAGTGGTGGCCTAACTACGGCTACACTAGAAGGACAGTATTTGGTATCTGCGCTCTGCTGAAGCCAGTTACCTTCGGAAAAAGAGTTGGTAGCTCTTGATCCGGCAAACAAACCACCGCTGGTAGCGGTGGTTTTTTTGTTTGCAAGCAGCAGATTACGCGCAGAAAAAAAGGATCTCAAGAAGATCCTTTGATCTTTTCTACGGGGTCTGACGCTCAGTGGAACGAAAACTCACGTTAAGGGATTTTGGTCATGAGATTATCAAAAAGGATCTTCACCTAGATCCTTTTAAATTAAAAATGAAGTTTTAAATCAATCTAAAGTATATATGAGTAAACTTGGTCTGACAGTTACCAATGCTTAATCAGTGAGGCACCTATCTCAGCGATCTGTCTATTTCGTTCATCCATAGTTGCCTGACTCCCCGTCGTGTAGATAACTACGATACGGGAGGGCTTACCATCTGGCCCCAGTGCTGCAATGATACCGCGAGACCCACGCTCACCGGCTCCAGATTTATCAGCAATAAACCAGCCAGCCGGAAGGGCCGAGCGCAGAAGTGGTCCTGCAACTTTATCCGCCTCCATCCAGTCTATTAATTGTTGCCGGGAAGCTAGAGTAAGTAGTTCGCCAGTTAATAGTTTGCGCAACGTTGTTGCCATTGCTACAGGCATCGTGGTGTCACGCTCGTCGTTTGGTATGGCTTCATTCAGCTCCGGTTCCCAACGATCAAGGCGAGTTACATGATCCCCCATGTTGTGCAAAAAAGCGGTTAGCTCCTTCGGTCCTCCGATCGTTGTCAGAAGTAAGTTGGCCGCAGTGTTATCACTCATGGTTATGGCAGCACTGCATAATTCTCTTACTGTCATGCCATCCGTAAGATGCTTTTCTGTGACTGGTGAGTACTCAACCAANTCATTCTGAGAATAGTGTATGCGGCGACCGAGTTGCTCTTGCCCGGCGTCAATACGGGATAATACCGCGCCACATAGCAGAACTTTAAAAGTGTTCATCATTGGAAAACGTTCTTCGGGGCGAAAACTCTCAAGGATCTTACCGCTGTTGAGATCCAGTTCGATGTAACCCACTCGTGCACCCAACTGATCTTCAGCATCTTTTACTTTCACCAGCGTTTCTGGGTGAGCAAAAACAGGAAGGCAAAATGCCGCAAAAAAGGGAATAAGGGCGACACGGAAATGTTGAATACTCATACTCTTCCTTTTTCAATATTATTGAAGCATTTATCAGGGTTATTGTCTCATGAGCGGATACATATTTGAATGTATTTAGAAAAATAAACAAATAGGGGTTCCGCGCACATTTCCCCGAAAAGTGCCACCTAAATTGTAAGCGTTAATATTTTGTTAAAATTCGCGTTAAATTTTTGTTAAATCAGCTCATTTTTTAACCAATAGGCCGAAATCGGCAAAATCCCTTATAAATCAAAAGAATAGACCGAGATAGGGTTGAGTGTTGTTCCAGTTTGGAACAAGAGTCCACTATTAAAGAACGTGGACTCCAACGTCAAAGGGCGAAAAACCGTCTATCAGGGCGATGGCCCACTACGTGAACCATCACCCTAATCAAGTTTTTTGGGGTCGAGGTGCCGTAAAGCACTAAATCGGAACCCTAAAGGGAGCCCCCGATTTAGAGCTTGACGGGGAAAGCCGGCGAACGTGGCGAGAAAGGAAGGGAAGAAAGCGAAAGGAGCGGGCGCTAGGGCGCTGGCAAGTGTAGCGGTCACGCTGCGCGTAACCACCACACCCGCCGCGCTTAATGCGCCGCTACAGGGCGCGTCCCATTCGCCATTCAGGCTGCGCAACTGTTGGGAAGGGCGATCGGTGCGGGCCTCTTCGCTATTACGCCAGCTGGCGAAAGGGGGATGTGCTGCAAGGCGATTAAGTTGGGTAACGCCAGGGTTTTCCCAGTCACGACGTTGTAAAACGACGGCCAGTGAGCGCGCGTAATACGACTCACTATAGGGCGAATTG

^#^: *Cryptococcus den*eo*formans* U6 promoter [1-280 nucleotides (nt)]; gRNA scaffold (305-380 nt); TEF promoter (389-1205 nt); 3x FLAG (1209-1274 nt); SV40 NLS (1281-1301 nt); Cas9 endonuclease (1302-5408 nt).
